# Relationship between perceived physical literacy and physical activity levels among Hong Kong adolescents

**DOI:** 10.1371/journal.pone.0203105

**Published:** 2018-08-27

**Authors:** Siu Ming Choi, Raymond Kim Wai Sum, Elean Fung Lin Leung, Robert Siu Kuen Ng

**Affiliations:** 1 Department of Sports Science and Physical Education, The Chinese University of Hong Kong, Shatin, NT, Hong Kong SAR, China; 2 Physical Education Unit, The Chinese University of Hong Kong, Shatin, N.T., Hong Kong SAR, China; University of Maiduguri College of Medical Sciences, NIGERIA

## Abstract

This study explores the relationship between the perceived physical literacy (PL) and physical activity (PA) levels of Hong Kong adolescents by using a cross-sectional study design. A total of 1,945 adolescents aged between 12 and 18, (1,028 male and 917 female) with a mean age of 14.98 (±1.65 years), took part in this study. A Perceived Physical Literacy Instrument (PPLI) and an International Physical Activity Questionnaire for Adolescents (IPAQ-A) were distributed to the participants within the first 15 minutes of PE lessons at their schools. The correlation between perceived PL and PA levels was low but significant (r = 0.227, p < 0.01), as was the correlation between the attributes of PL and PA intensity and the domains of PA (r = 0.067–0.292, p < 0.01). A significant linear equation was computed (F (3, 1941) = 35.679, p < 0.01), with an R^2^ of 0.052. The metabolic equivalent (MET) minutes representing participants’ predicted PA levels were –5490 + 366.1 (sense of self and self-confidence) + 221.866 (self-expression and communication with others) + 287.748 (knowledge and understanding). Looking at individual factors, the correlation between perceived PL and PA levels showed no significant difference across gender (r _male_ = 0.234; r _female_ = 0.198) but showed a significant difference across grade level (r _junior_ = 0.302; r _senior_ = 0.197), school bands (r _band 1_ = 0.31; r _band 2_ = 0.263; r _band 3_ = 0.191) and socio-economic status (SES) (r_low_ = 0.225; r_medium_ = 0.35; r_high_ = 0.191). The relationship between perceived PL and PA levels was significantly low but was closely related to the recreational PA, including individual factors such as gender, grade levels, school band and SES. Future studies could focus on school-based PA intervention programmes for perceived PL and the relationship between perceived PL and objective PA levels.

## Introduction

Physical literacy (PL) is defined as ‘the motivation, confidence, physical competence, knowledge, and understanding to value and take responsibility for engaging in physical activities for life’ [1]. Specifically, an individual should be able to demonstrate the interaction between the physical nature of movement by using their experiences and knowledge in other environments [2–4]. In short, a physically literate individual is a motivated, skilful and confident person who will participate in physical activity (PA) throughout his/her life. The World Health Organization defines PA as ‘any bodily movement produced by skeletal muscles that requires energy expenditure’ [5]. The health benefits of engaging in a physically active lifestyle are linked with public health goals such as controlling weight, reducing chronic diseases (e.g., cardiovascular disease, type II diabetes, cancer), and strengthening bones and muscles [4–6]. PA guidelines recommend that adolescents participate in moderate to vigorous physical activity (MVPA) for at least 60 minutes per day [5, 6].

Some Western countries already promote PL through PA in their schools, communities and public health organisations [7]. The Australian government has allocated A$200 million to developing and implementing PL-oriented PA programmes [8]. In Hong Kong, the concept of PL has been promoted to Physical Education (PE) teachers through Continuing Professional Development (CPD) held by the Education Bureau (2015). However, schools are only required to allocate 5–8% of total lesson time (70–80 minutes per week) to PE lessons [9], which falls short of the standard in other countries, where it averages 100 minutes per week [10]. Although the duration of PE lessons does not reflect adolescents’ overall PA levels, recent local studies have found that 57.7% and 18.3% of Hong Kong adolescents are physically inactive [11] and sedentary [12], respectively, which negatively affects their health [13]. The participants in these studies claimed that a lack of spare time, fatigue and laziness were the main reasons for not attempting PA. To promote the benefits of achieving the worldwide PA recommendation, the Guidelines of Quality of Physical Education (QPE) state that PL is the foundation of PE, and the development of PL is crucial for adolescents in encouraging adolescents to participate in lifelong PA [4].

Physically literate individuals will likely participate in lifelong PA, as they appreciate the intrinsic value of PE in contributing to their health [4, 14]. Studies have discovered that ‘PL is an antecedent of PA while also being developed through PA’ [15]. In other words, individuals might not participate in PA without the understanding of PL, yet through participating in PA, they can develop as physically literate individuals. In the Canadian preview on PL, they explained that there is an inter-relationship between PL and PA [16], especially in the sense that PL is the fundamental concept of PE [4]. Physically literate adolescents can gain self-confidence in fundamental movement, coordination and control in their changing environments through PA. This will lead them to be more confident in verbal and non-verbal communication and help them to enjoy discovering new activities.

Studies on PL and PA have focused on how PE educators, curriculum and pedagogy or countrywide interventions advocate PA to promote PL, but few have investigated PL and its related constructs. Because no instrument exists to measure PL and PA directly, this study used the perceived PL and PA levels, which represented how adolescents perceived their physically literacy, to value and engage with PA. It used PA levels to represent the adolescents’ behaviour and exercise intensities [17–19] in investigating adolescents’ perceived PL and PA levels. PL was proposed to be associated with individual factors such as gender, grade level [17], and social and environmental variables (e.g. socioeconomic status, SES) [15], which also affected the relationship between PL and PA.

## Method

### Study design

The relationship between perceived PL and PA levels was investigated through a cross-sectional study of adolescents studying in various secondary schools in Hong Kong. Scores for perceived PL and time spent on PA were self-reported by the participants and recorded with a set of questionnaires using the Perceived Physical Literacy Instrument (PPLI) and the International Physical Activity Questionnaire for Adolescents (IPAQ-A). The demographic information of participants such as gender, grade levels, school bands and SES were also collected. Approval for the use of human subjects was obtained from the University Survey and Behavioural Research Ethics Committee of the Faculty of Education, the Chinese University of Hong Kong.

### Participants

A stratified sample of urban and suburban schools was used in this study. A letter of invitation was sent to schools. In each school that agreed to participate in the study, two classes were randomly selected in each grade. Parental consent was obtained before the data collection process. Five urban and suburban secondary schools were recruited, and 2,089 adolescents aged between 12 and 18 participated in the study, 1,945 responses were completed and considered as valid data for further analysis. The mean age was 14.98 (±1.65 years), and there were 1,028 males and 917 females’ participants. Questionnaires were distributed to the participants within the first 15 minutes of their school PE lessons. The questionnaires asked about perceived PL and how much time the participants had dedicated to PA during the previous week.

### Self-report measures

#### Physical literacy

The PPLI was constructed from a literature review on the concept of PL covering 1994 to 2015, followed by three focus group interviews in which 11 Hong Kong PE teachers shared their perceptions of PL and provided diverse experiences on how PL impacts their students’ lifestyles. It is a 9-item instrument used to measure both students’ and teachers’ perceived PL. It includes three sub-scales: ‘sense of self and self-confidence’, ‘self-expression and communication with others’ and ‘knowledge and understanding’ which are identified as key attributes of Whitehead’s concept of PL. The participants responded on a 1 to 5-point Likert scale (1 = strongly disagree; 5 = strongly agree). Example statements include: ‘I am physically fit, in accordance with my age’ (sense of self and self-confidence), ‘I have strong social skills’ (self-expression and communication with others) and ‘I am aware of the benefits of sports related to my health’ (knowledge and understanding). The scale scores were reliable, with an internal consistency ranging from .73 to .76, which supports factorial validity [20].

#### Physical activity

The IPAQ-A was modified a version of the IPAQ used to investigate the validity of adolescents’ self-reported PA levels using accelerometer data. The IPAQ-A was validated using Spearman’s rank correlation (ρ = 0.17–0.30, p<0.05) with accelerometer data in healthy European adolescents. It was carefully adapted based on interviews and experience from the study centres, and accelerometer data were considered for concurrent validity [21]. The IPAQ-A has also been used to investigate the relationship between PA and VO_2_ max among European adolescents [22]. The following shows the IPAQ’s modification into the IPAQ-A, which was used to validate the accelerometer data.

The IPAQ-A involves four environmental domains: school-related, housework and gardening, transportation and recreation, and sport and leisure time. These domains are used to capture participants’ walking activity and moderate or vigorous PA performed for at least 10 uninterrupted minutes in the previous 7 days (5 school days and 2 weekend days). IPAQ questions about PA in the work domain were replaced by the school domain to assess activities such as PE lessons, breaks and MVPA. The housework domain was amended to only contain one question focused on PA at home, which could include carrying heavy loads, scrubbing floors and sweeping, which require at least moderate physical effort and make the participant breathe harder than normal [21]. The order of the PA levels in the transportation and recreation domain was changed to place walking first, followed by vigorous and moderate activities, to avoid over-reporting [22, 23]. The data were collected in minutes per week (min/week) for each of the PA intensities. MET values were calculated using the formula for computation of MET-minutes [24, 25].

#### Demographic information

The participants’ demographic information, such as gender, grade level, school band and SES, was also collected. In Hong Kong, secondary school students are divided into three school bands—bands 1, 2 and 3 in accordance with their academic achievement. The scaled internal and external assessment marks of all students were ordered. Then they were equally divided into three school bands and allocated to secondary schools, each consisting of one third of the total number of students [26]. For the SES, monthly household incomes of HK$ 0–14,999, HK$15,000–29,999 and HK$>30,000 denoted the low-, medium- and high-income group, respectively. The medium-income group earns the median monthly domestic household income [27].

### Statistical analysis

Pearson’s Product-Moment Correlation was used to compute the correlations between the attributes of perceived PL and PA levels. Multiple regression analysis was used to examine the association between the perceived PL and PA levels of those participants. Descriptive statistics of the participants’ demographic information, such as gender, grade level (junior and senior secondary), school band and SES were considered in the Pearson’s Product-Moment Correlation to compute the correlation between perceived PL and PA levels. The z-scores between the correlations were computed to examine whether those samples were significantly different [28]. SPSS version 22 for Windows was used for the data analysis. The confidence level was set as 95% (P ≤ 0.05) to maintain statistical reliability.

## Results

The adolescents reported their perceived PL on average as 10.70 (±2.22), 10.80 (±2.11) and 12.01 (±1.90) for the three components, ‘sense of self and self-confidence’, ‘self-expression and communication with others’ and ‘knowledge and understanding’, respectively. Their average daily PA time and intensity was 74 minutes at a walking pace, with 34 minutes at a moderate pace and 27 minutes at a vigorous pace, which met the guidelines recommending at least 60 minutes per day of participation in any MVPA (61 minutes). The time spent in different PA domains was on average, 47 minutes at school, 17 minutes at home, 66 minutes during transportation and 48 minutes during recreation.

Table 1 shows the significant correlations between the perceived PL and PA levels (r = 0.227, p < 0.01) of the Hong Kong adolescents measured by the PPLI and IPAQ-A. Although the correlations are modest, components of perceived PL, intensities of PA levels and three domains (school, transportation, and recreation) of PA were significantly correlated (r = 0.067–0.292, p < 0.01). No significant correlation was found for the home domain.

**Table 1:** Pearson’s Product-Moment Correlation (r) of components of perceived PL and intensity and PA domains (N = 1945).

	Total PA	Walking	Moderate	Vigorous	School	Home	Transport	Recreation
Total PL	0.227^a^	0.096^a^	0.202^a^	0.203^a^	0.102^a^	0.033	0.098^a^	0.292^a^
Sense of self and self-confidence	0.205^a^	0.081^a^	0.173^a^	0.189^a^	0.094^a^	0.011	0.082^a^	0.267^a^
Self-expression and communication with others	0.166^a^	0.086^a^	0.180^a^	0.132^a^	0.069^a^	0.077^a^	0.091^a^	0.201^a^
Knowledge and understanding	0.185^a^	0.069^a^	0.141^a^	0.177^a^	0.086^a^	-0.010	0.067^a^	0.247^a^

^*a*^ *Correlation is significant at the 0*.*01 level (2-tailed)*.

A multiple linear regression was calculated to predict the PA levels of participants based on their perceived PL attributes. Preliminary analyses were performed to ensure no violations of the assumptions of normality, linearity and multicollinearity. Table 2 shows the significant regression equation (F (3, 1941) = 35.679, p < 0.01), with an R^2^ of 0.052. The participants predicted PA levels were –5490 + 366.1 (sense of self and self-confidence) + 221.866 (self-expression and communication with others) + 287.748 (knowledge and understanding) MET minutes when the attributes of perceived PL were measured on a 5-point Likert Scale. The results showed that sense of self and self-confidence (β = 0.123, *p* = 0.000), self-expression and communication with others (β = 0.071, *p* = 0.003) and knowledge and understanding (β = 0.082, *p* = 0.007) were positively related to PA level. The participants’ mean PA levels increased by 366.1, 22.1866 and 287.748 MET minutes for each attribute score of perceived PL. All attributes of perceived PL were significant predictors of PA level.

**Table 2:** Results of multiple regression analysis predicting PA level using perceived PL attributes (N = 1945).

Variable	B ^a^	SEB ^b^	β ^c^	*p* ^d^
Constant	–5490	1016.41	-	0.000
Sense of self and self-confidence	366.1	85.771	0.123	0.000
Self-expression and communication with others	221.866	81.515	0.071	0.003
Knowledge and understanding	287.748	97.537	0.082	0.007
R = 0.229	-	-	-	-
R^2^ = 0.052	-	-	-	-
ΔR^2^ = 0.052	-	-	-	-

^a^ Unstandardized coefficient and constant for the linear regression equation.

^b^ Standard Error of B.

^c^ Standardized regression coefficient.

^d^ Probability value.

The research team then explored the factors of gender, grade level, school band and SES and their influence on the relationship between perceived PL and PA levels, as shown in Table 3. All groups showed significant positively correlations between perceived PL and PA.

**Table 3:** Pearson’s Product-Moment Correlation (r) and z-scores of perceived PL and PA levels across factors.

	Gender		Grade level		Academic achievement by school band			Socio-economic status		
	Male	Female	Junior secondary	Senior secondary	Band 1	Band 2	Band 3	0–14999	15000–29999	>30000
N	1028	917	1227	717	1252	229	464	589	813	543
r	0.234^a^	0.198^a^	0.302^a^	0.193^a^	0.310^a^	0.263^a^	0.191^a^	0.225^a^	0.350^a^	0.191^a^
z	0.238	0.201	0.312	0.195	0.321	0.269	0.193	0.229	0.365	0.193

^a^ Correlation is significant at the 0.01 level (2-tailed).

To determine whether the samples were significantly different, the z-scores and their differences were computed, as shown in Tables 3 and 4, respectively. Although the correlation between the perceived PL and PA levels of males (r = 0.234) was higher than that for females (r = 0.198), no significant difference was found in gender. For grade level, junior secondary adolescents obtained a higher correlation (r = 0.302) than senior secondary adolescents (r = 0.193), and the difference was significantly. For the school band factor, band 1 adolescents attained the strongest correlation between perceived PL and PA levels (r = 0.191), followed by band 2 (r = 0.263) and band 3 (r = 0.191), while only band 1 and 3 were significantly different. Students from the medium-income group had a higher correlation between perceived PL and PA levels (r = 0.35) than the low-income group (r = 0.225) and the high-income group (r = 0.191). The medium-income group was significantly different from the others.

**Table 4:** Z-score differences between perceived PL and PA Levels across factors.

	Gender	Grade levels	Academic achievement by school band			Socio-economic status		
	Male and female	Junior and senior secondary	Band 1 and 2	Band 1 and 3	Band 2 and 3	Low and medium	Low and high	Medium and high
z-difference	0.83	2.47	0.71	2.33	0.94	–2.52	0.6	3.1
p	0.4065	0.0135^b^	0.4777	0.0198^b^	0.3472	0.0117^b^	0.5485	0.0019^a^

^a^ Correlation is significant at the 0.05 level (2-tailed).

^b^ Correlation is significant at the 0.01 level (2-tailed).

## Discussion

In this study, we discovered a relationship between perceived PL and PA levels, and how individual factors and SES relate to this relationship. Longmuir and Tremblay [17] presented one of the top future research topics for PL: researchers should not only identify the effects of individual factors on PA behaviour, but also how they affect Whitehead’s [14] six components, to understand and enhance lifelong PL. The six components are ‘sense of self and self-confidence’, ‘self-expression and communication with others’, ‘knowledge and understanding’, ‘motivation’, ‘confidence and physical competence’, and ‘interaction with the environment’. There was previously no instrument for perceived PL, but the PPLI was constructed and validated to measure perceived PL [20]. Using this instrument, we could discover the relationship between the components of perceived PL and PA levels.

### Relationship between PL and PA

Forty-four percent of relevant research papers indicate that PA is central to PL, and without PL, individuals would not participate in PA [15]. Longmuir and Tremblay [17] noted that ‘PL is not something one does or does not have, or does or does not achieve. Rather, it is the individual’s lived experience of PA’. In this study, we used perceived PL and PA levels to show whether adolescents were physically literate by judging their value of and participation in PA, and analysing their PA levels through their behaviour and different PA intensities. Thus, the relationship between perceived PL and PA levels actually compared their ‘lived experience of PA’ with respect to their perceived PL. However, significant weak and positive correlations between perceived PL and PA levels were recorded in this study, which was inconsistent with the previous literature. The low positive correlation could be explained by the adolescents perceiving their PL as moderate but could not matching this with their high levels of PA. As previously mentioned, PL is a new concept in Hong Kong, and PE teachers may not have had enough time to transfer it to their students.

This study also demonstrated the predictive effect of PL attributes on PA levels among Hong Kong adolescents using the multiple regression analysis. From the attributes of sense of self and self-confidence, adolescents can gain rewarding PA experiences to develop on their PL journey. Through verbal and non-verbal communication, the attributes of self-expression and communication with others can encourage them to react to interpersonal interactions through feeling and responding while participating in PA. The knowledge and understanding attributes are held by individuals who can describe and evaluate their movement experiences and PA lifestyle and how to improve their movement and all-round health [14]. These PL attributes (self and self-confidence, self-expression and communication with others and knowledge and understanding) generated a variance of 5.2% in PA levels. The concept of PL should be introduced to adolescents by assigning them the responsibility of designing their chosen PA and discussing the importance of PA for lifelong health. Such experiences can help them understand the value of PA and realise that they are responsible for maintaining it [18, 19].

### Relationships between PL, MVPA and recreational PA

Previous scholars have focused on measuring objective PA levels, but this does not reflect perceived PL [17]. There is a need to determine whether perceived PL is associated with different PA intensities and domains. As previously stated, worldwide PA guidelines recommend that individuals participate in daily MVPA for at least 60 minutes. Although Hui [12] and Ha et al. [13] concluded that PA levels among Hong Kong adolescents were not high enough to promote health, the participants in this study met the global MVPA guidelines, which also relate to a moderate score for perceived PL. In this sense, they could distinguish between walking intensity and both moderate and vigorous physical effort.

Although maximising school-based PA helps adolescents to develop PL competencies by boosting their MVPA and building on their intrinsic motivation to engage in PA, promoting the concept of PL is not solely the responsibility of PE teachers [29]. Teaching this concept is not restricted to PE lessons alone. Individuals can develop PL through coaches, family members and peers [30], and this can help to influence and shape their lifelong journeys [15]. Whether the experience is positive or negative, it can still influence adolescents’ understanding of what it means to be physically literate. It is important that the fundamental components of PL are enhanced and nurtured during adolescence [18, 19]. In addition to structured or unstructured PA at school, adolescents can choose recreational PA at their preferred level of intensity. Students have more freedom to choose their recreational PA. During recreational PA, parents, coaches and peers can also affect adolescents’ lifelong PA journeys. In our study, free-choice recreational PA correlating with perceived PL was significantly preferred over school (z-difference = 6.18), home (z-difference = 8.34) and transportation (z-difference = 6.31) PA (p < 0.05). Without school restrictions on guidelines and curriculum, adolescents can choose to participate in activities organised by sports clubs and community centres, or on their own. During adolescence, individuals need to respond to the challenges they encounter, including acute self-consciousness and fear of embarrassment. By nurturing adolescents’ PL through recreational PA, practitioners and adolescents gain confidence in themselves and develop good habits that will encourage them to participate in lifelong PA [18]. Adolescents’ recreational PA is geared towards activities that interest them with active encouragement from practitioners, so it naturally obtained a higher correlation between perceived PL and their PA levels in this study.

### Individual factors and SES’ effect on the relationship between PL and PA

Scholars have proposed that PL is associated with gender, grade level [17] and SES [15] which are also factors in the relationship between perceived PL and PA levels. In this section, the situations of and differences between each group related to this relationship will be discussed.

A previously cited citywide survey found a gender gap in PA, with 49.9% of males and 65.8% of females defined as physically inactive [11]. Female adolescents are more likely to drop out of PA, which halts their progress with PL. It is thus important to support them in participating in PA in line with their interests. A case study showed that organising PA according to students’ preferences and having better communication between students and practitioners would increase students’ PL [31]. It also discovered that the correlations between the perceived PL and PA levels of males and females were not significantly different and the intensity, quality and quantity of PE lessons and sports clubs were the same [32, 33]. Male adolescents participated more in PA in higher intensity sports and exercises (ball games), while female adolescents engaged more in low- and medium-intensity exercise, such as walking, swimming and roller/ice skating [11, 33]. However, all of the participants made progress on their own PL journeys by participating in their preferred PA.

PA is important at all ages, for fundamental movement and coordination, cognitive development, and peer and social support [34]. Without PL knowledge and participation in PA, student development will be limited. A significantly difference was found in the correlations between the perceived PL and PA of adolescents studying at junior and senior secondary levels. Ingram [35] stated that the decline in PA with age has a negative linear association with a strong biological basis, and Dmitruk et al. [36] discovered that the PA levels of European students decreased with age. Compared to senior adolescents, junior adolescents receive less academic pressure from public examinations. This may increase the time they have available for PA, which may help them to develop a lifelong PL and acquire a stronger relationship between perceived PL and PA levels.

Keeley and Fox [37] investigated the positive physiological mechanism between PA and cognition and concluded that there is a potential relationship between PA and academic achievement in children [38]. Other scholars further investigated the positive relationship between PA and academic achievement [38–42]. Usually, adolescents with higher academic ability in Hong Kong are enrolled in band 1 schools. The physiological and psychological mechanisms of PA and academic achievement showed an increased brain-derived neurotrophic factor and higher self-esteem in band 1 students [38]. Therefore, students with a higher capacity to learn might more easily understand how a new concept in a PE context can affect physically literate individuals and that how they participate in PA can bring them positive health effects.

Tomporowski et al. [34] found that families of different SES participated in different types of sport, exercise and PA. Research from other countries (the USA, Poland, Italy, and Finland) has also supported the positive relationship between SES and PA or extra-curricular activities [36, 40, 42]. Higher-SES families can afford to give their children more opportunities. They can afford class fees and equipment, and drive their children to and from venues–all of which increase the probability that their children will be physically active [36] and more physically literate [34]. Students from families with a monthly income of HK$ 15,000–29,999 (the median monthly domestic household income group) [27] obtained a stronger correlation than those in other income groups in this study. A global PA survey revealed that it is more common for individuals in higher-income countries to participate in less PA than those in lower-incomes countries [43]. In this regional study, when the research group split individuals into three income groups, the middle-income group had a stronger relationship between perceived PL and PA level than the lower- and higher-income groups. As PA was composed of four domains in the IPAQ-A, different income groups participated in different PA domains, with people from the lower-income group participating less frequently in recreation, and vice versa [34, 40, 42]. Social patterns may be changing [43], and this study indicates that middle-income families appear to encourage adolescents to participate in more PA through monetary or non-monetary support [36].

### Limitations

This study has several limitations. The measured PL was perceived by the adolescents themselves, making it unobservable. Self-reported PPLI is one method to identify perceived PL in a way that is consistent with using the self-reported IPAQ-A. It is well-known that the IPAQ-A is easy to over-report, as the meaning of the PA domains is often misunderstood by participants [43]. There are several ways to improve the data collection process. For instance, our team provided examples for each statement or question, which may have encouraged participants to finish the questionnaire. In addition, the design of the IPAQ-A makes it prone to error, leading some participants to miss some questions. This might invalidate the questionnaire or add noise to the data, leading to a high standard deviation of PA levels. Accordingly, the IPAQ-A cannot generate meaningful analysis of covariance results. Instead, we only used z-score differences to assess covariance. However, as the IPAQ-A is a globally standardised and validated tool, it is still applicable for collecting self-reported PA levels.

## Conclusion

This study is important due to the lack of research on the relationship between perceived PL and PA levels using a cross-sectional study design. The attributes of perceived PL and PA levels were associated with recreational PA and individual factors such as gender, grade level, school band and SES. The concept of PL has been introduced to PE teachers in Hong Kong in CPD workshops. This study indicates the relationship between PL and PA if the concept of PL could be taught as part of the curriculum and PA levels therefore became part of students’ learning outcomes. By exploring the relationship between perceived PL and PA levels, the findings supported the importance of PL development in PE lessons in Hong Kong and PE teachers’ assessment of students’ self-perception of PL and PA levels. This approach will also enhance students’ learning and the effectiveness of teaching through the re-design of the PE curriculum.

The findings of this study will be useful in any future study determining the perceptions of PL and participation in PA for adolescents in Hong Kong using randomised controlled trials in school-based PA programmes or recreational PA. We could focus on self-reported perceived PL and objective measures of PA levels to more precisely evaluate the relationship. Ultimately, by using more advanced study designs and statistical methods, researchers could generate a meaningful and measurable relationship between perceived PL and PA levels and develop practical intervention programmes for different stages of an individual’s journey to achieving lifelong PL.
